# On the Transient Queue with the Dropping Function

**DOI:** 10.3390/e22080825

**Published:** 2020-07-28

**Authors:** Andrzej Chydzinski

**Affiliations:** Department of Computer Networks and Systems, Silesian University of Technology, Akademicka 16, 44-100 Gliwice, Poland; andrzej.chydzinski@polsl.pl

**Keywords:** queueing model, dropping function, M/G/1, Internet routers, active queue management, transient analysis

## Abstract

We deal with a queueing system, in which arriving packets are being dropped with the probability depending on the queue size. Such a scheme is used in several active queue management schemes proposed for Internet routers. In this paper, we derive and analyze a selected transient characteristic of the model, i.e., the probability that in a given time interval the queue size is kept under a predefined level. As the main purpose of the discussed queueing scheme is to maintain the queue size low, this is a natural characteristic to study. In addition to that, the average time to reach a given level is derived. Theoretical results for both characteristics are accompanied by numerical examples. Among other things, they demonstrate that the transient behavior of the queue may vary significantly with the shape of the dropping function, even if the steady-state performance remains unaltered.

## 1. Introduction

It is known that there is a partial divergence in the design goals of the network layer and the transport layer in the contemporary TCP/IP networks and the Internet. Namely, the buffers in the network layer (routers) were originally meant to store bursts of packets, occurring occasionally due to statistical multiplexing of different flows. Such buffers do not have to be large, and they do not introduce a substantial queueing delay. On the other hand, developed much later, congestion control for the transport layer uses the same buffers for a different purpose. Namely, the TCP protocol probes the throughput, currently available for a particular flow, by increasing its sending rate, until the buffer is full and something bad happens (e.g., a packet is lost or the queueing delay gets large). Such behavior of TCP makes the buffers overflowed for a significant fraction of time, no matter how large they are. As a consequence, the queueing delays are unnecessarily high. This is the so called bufferbloat phenomenon, well described in the networking literature, for example, [1,2].

As a cure for this, the Internet Engineering Task Force advises the application of active queue management in Internet routers, [3]. The general idea is that every packet arriving to the router can be dropped with some probability. Roughly speaking, this probability should be relatively high, when there are signs of forthcoming congestion. Such preventive packet dropping enables a substantial reduction of the average queue size and delay, and has other positive effects (e.g., desynchronization of TCP flows). The dropping probability, however, cannot be too high, as emptying the buffer completely would cause an underutilization of the link.

How exactly the dropping probability should evolve and on which factors it should depend, is a long debate among researchers. Several advanced algorithms for computing the dropping probability were proposed to date, see, for example [4,5,6,7] and the references given there. Some propositions are based on artificial neural networks (e.g., [8]), fuzzy logic (e.g., [9]) or genetic algorithms (e.g., [10]). In others, the dropping probability is replaced by a deterministic decision about each arriving packet, i.e., whether to drop it, or not [11].

A very important subclass of algorithms proposed in the literature exploits an idea of the dropping function. Namely, an arriving packet can be dropped with the probability being a function of the queue size. Several candidates for such function have been studied, beginning with the linear function [12], through the doubly-linear one [13], exponential [14], quadratic [15], cubic [16], and the newest proposition being a mixture of linear and cubic functions [17]. The algorithms based on the dropping function may not always provide as good performance as those cited in the previous paragraph, but their greatest advantage is an extreme ease of implementation, accompanied by a decent performance, much better than no active management at all. Obviously, to provide a theoretical background for such algorithms, queueing models with the dropping function have to be studied. Initially, they were mainly studied via simulations. Recently, more efforts have been made on mathematical analysis, using tools and concepts of the queueing theory.

In this paper, we derive a selected transient characteristic of the queueing model with the dropping function—the probability that in a given time interval the queue size is kept under a predefined level. As argued above, the main purpose of the discussed queueing scheme is to maintain a low queue size. Therefore, such characteristic is very natural to deal with. In addition to that, the average time to reach a given level is studied. The importance of those characteristics is underlined in numerical examples, where it is shown that different dropping functions may induce a very different transient behavior of the system, even if they provide the same stationary behavior.

We assume Poisson arrivals and general service time distribution in the analysis. The dropping function is also assumed in a general form, i.e., every mentioned above function (and many others) can be studied using the obtained results. In other words, the model studied herein is the M/G/1 model in Kendall’s notation, with the addition of the dropping function. Note that such a model is very general. It incorporates the classic M/G/1 model (when the dropping function equals 0 for every argument), the classic M/G/1/N model (when the dropping function assumes 1 for n≥N and 0 otherwise) and the M/G/1/N model with the dropping function (when the dropping function assumes 1 for n≥N and some other values otherwise).

To the best of the author’s knowledge, the results presented herein are new. For studies on other characteristics (the queue size, loss probability, response time) of systems with the dropping function, or carried out under different assumptions on the arrival process and service times, we refer the reader to [18,19,20,21,22,23,24,25,26,27,28]. On the other hand, there are several papers on the time to reach a given level in classic queueing models, i.e., without the dropping function—see, for example, [29,30,31,32,33] and the references given there.

The analytical method used herein is based on formulating and solving a system of Volterra integral equations (see also, e.g., [34,35]). In this method, the system of equations is formulated first using probabilistic properties of the model. Then it is solved using the Laplace transform technique. Finally, in order to perform numerical calculations, a method for inverting the Laplace transform is needed. We recommend one such method, which combines a good speed with a decent accuracy in inverting probability distributions.

The rest of the paper is structured as follows. In Section 2, the queueing model is formally presented and accompanied by basic notations and conventions. In Section 3, an analysis of the probability of not reaching a given level in a time interval, as well as the average time to reach a given level, is carried out. The analysis ends with formulas for the two characteristics, presented in Theorem 1 and Corollary 1, respectively. In the same section, computational aspects are discussed, with the emphasis on obtaining numerical values by using the transform inversion. In Section 4, numerical examples are shown. They are focused on demonstrating the impact of the dropping function on the studied transient characteristics. Namely, five different dropping functions are shown to induce different transient behavior, even if each of them is parameterized to provide the same stationary behavior (the average response time). Additionally, an example on how we may design the dropping function in such a way that it meets some performance goals, is presented. Finally, remarks concluding the paper are given in Section 5.

## 2. Model Description

We analyze the M/G/1 queueing model in Kendall’s notation, with the addition of the dropping function in a general form.

Namely, the arrival process is Poisson with the rate λ, while the service time has distribution function F(·), which is not further specified, except for the fact, that its average value is finite, i.e.,:

(1) $$\int_{0}^{\infty }tdF\left(t\right)<\infty .$$

The buffer for packets is infinite.

Moreover, each arriving packet can be dropped (deleted) with the probability d(n), where *n* is the length of the queue at the time of this packet arrival, including the packet being serviced, if applicable. The dropping function d(n) may assume any value in [0,1] for every n=0,1,2,…. The queueing discipline is irrelevant in the analysis, it can be FIFO, LIFO, etc.

The following notation will be used: P for probability, E for the average value of a random variable, X(t) for the queue length at the time *t*, including the service position, if occupied. We use the convention that X(t) is left-continuous, i.e., X(t)=X(t−). If X(0+)>0, then it is assumed that t=0 is the beginning of the service time.

## 3. Analysis

The main characteristic of interest, i.e., the probability that the queue size does not reach level *M* by the time *t*, assuming it starts from the level *n*, will be denoted by $Y_{n,M}\left(t\right)$, i.e.,:

(2) $$Y_{n,M}\left(t\right)=P\left(H_{n,M}>t\right),$$

where

(3) $$H_{n,M}=\inf\left\{t>0:X\left(t\right)=M|X\left(0+\right)=n\right\},\;\;\;0\leq n<M,$$

is the time of reaching the level *M*.

Our first goal is to derive the formula for the Laplace transform of the function $Y_{n,M}\left(t\right)$, i.e.:

(4) $$y_{n,M}\left(s\right)=\int_{0}^{\infty }e^{-st}Y_{n,M}\left(t\right)dt,\;\;\;s>0,$$

in the vector form:

(5) $$y_{M}\left(s\right)={\left[y_{0,M}\left(s\right),\ldots ,y_{M-1,M}\left(s\right)\right]}^{T}.$$

Let $Q_{n,k}\left(u\right)$ denote the probability, that in the time interval (0,u] exactly *k* packets were accepted to the queue, assuming that it was X(0+)=n and there was no service completion by the time *u*. In other words, $Q_{n,k}\left(u\right)$ is the number of effective arrivals in the interval (0,u], after the filtration by the dropping function. In addition to $Q_{n,k}\left(u\right)$, the following transforms will be of use:

(6) $$a_{n,k}\left(s\right)=\int_{0}^{\infty }e^{-su}Q_{n,k}\left(u\right)dF\left(u\right),\;\;\;s>0,$$

(7) $$b_{n,k}\left(s\right)=\int_{0}^{\infty }e^{-su}Q_{n,k}\left(u\right)\left(1-F\left(u\right)\right)du,\;\;\;s>0.$$

Using the law of total probability with respect to the first departure time *u*, for 1≤n≤M−1 we have:

(8) $$Y_{n,M}\left(t\right)=\sum_{k=0}^{M-n-1}\int_{0}^{t}Q_{n,k}\left(u\right)Y_{n+k-1,M}\left(t-u\right)dF\left(u\right)+\left(1-F(t)\right)\sum_{k=0}^{M-n-1}Q_{n,k}\left(t\right).$$

In particular, the first part of (8) corresponds to the event that the first departure happens before *t*. In this case, the number of effective arrivals by the time *u* must be less than M−n. At the first departure time, *u*, the new queue size is clearly n+k−1, while the remaining time to hit the level *M* is t−u. The second part of (8) corresponds to the event that the first departure happens after *t*, which has probability 1−F(t). In this case, the number of effective arrivals must be less than M−n by the time *t*.

For n=0, we can use the law of total probability with respect to the first arrival time, *v*. Namely, we obtain:

(9) $$Y_{0,M}\left(t\right)=\left(1-d(0)\right)\int_{0}^{t}Y_{1,M}\left(t-v\right)\lambda e^{-\lambda v}dv+d\left(0\right)\int_{0}^{t}Y_{0,M}\left(t-v\right)\lambda e^{-\lambda v}dv+e^{-\lambda t}.$$

The first part of (9) corresponds to the case where a packet arrives by the time *t* and it is accepted to the system. Therefore, the new queue size at the time *v* is 1 and the remaining time to hit the level *M* is t−v. The second part of (9) corresponds to the case where a packet arrives by the time *t*, but it is dropped. Therefore, the queue size is still 0 at the time *v*. Finally, the third part of (9) corresponds to the case where there are no new arrivals by the time *t*. It means also that the queue size cannot hit the level *M* by the time *t*.

Applying the Laplace transform to (8) and (9), we get:

(10) $$y_{n,M}\left(s\right)=\sum_{k=0}^{M-n-1}a_{n,k}\left(s\right)y_{n+k-1,M}+\sum_{k=0}^{M-n-1}b_{n,k}\left(s\right),\;\;\;\;1\leq n\leq M-1,$$

and

(11) $$y_{0,M}\left(s\right)=\frac{\lambda \left(1-d(0)\right)}{s+\lambda }y_{1,M}\left(s\right)+\frac{d(0)\lambda }{s+\lambda }y_{0,M}\left(s\right)+\frac{1}{s+\lambda },$$

respectively.

Now, introducing the matrix $R_{M}\left(s\right)$ defined as:

(12) $$R_{M}\left(s\right)={\left[r_{i,j}\left(s\right)\right]}_{i=0\ldots M-1,j=0\ldots M-1},$$

(13) $$\begin{array}{cc} \; & r_{i,j}\left(s\right)=\left\{\begin{array}{c} 1-\frac{d(0)\lambda }{s+\lambda },\;\;\;\;\mathrm{if}\;\;i=j=0,\\ \frac{(d(0)-1)\lambda }{s+\lambda },\;\;\;\;\mathrm{if}\;\;i=0,j=1,\\ a_{i,0}\left(s\right),\;\;\;\;\mathrm{if}\;\;i=j+1,\\ a_{i,1}\left(s\right)-1,\;\;\;\;\mathrm{if}\;\;1\leq i=j\leq M-2,\\ a_{i,j-i+1}\left(s\right),\;\;\;\;\mathrm{if}\;\;1\leq i<j\leq M-2,\\ -1,\;\;\;\;\mathrm{if}\;\;i=j=M-1,\\ 0,\;\;\;\;\mathrm{otherwise}, \end{array}\right. \end{array}$$

and vector:

(14) $$w_{M}\left(s\right)={\left[w_{0,M}\left(s\right),\ldots ,w_{M-1,M}\left(s\right)\right]}^{T},$$

(15) $$w_{0,M}\left(s\right)=\frac{1}{s+\lambda },$$

(16) $$w_{i,M}\left(s\right)=-\sum_{k=0}^{M-i-1}b_{i,k},\;\;\;1\leq i\leq M-1,$$

from (10) and (11) we have:

(17) $$R_{M}\left(s\right)y_{M}\left(s\right)=w_{M}\left(s\right).$$

Finally, from (17) we obtain the following theorem.

**Theorem** **1.** *The Laplace transform of the probability that the queue size does not reach the level M by the time t is:*

(18) $$y_{M}\left(s\right)=R_{M}^{-1}\left(s\right)w_{M}\left(s\right),$$

*where the matrix $R_{M}\left(s\right)$ is given in (13), while the vector $w_{M}\left(s\right)$ in (15) and (16).*

It easy to see that:

(19) $$EH_{n,M}=\int_{0}^{\infty }P\left(H_{n,M}>t\right)dt=\int_{0}^{\infty }Y_{n,M}\left(t\right)dt=\lim_{s\to 0+}y_{n,M}\left(s\right).$$

Thus we have the following corollary.

**Corollary** **1.** *The average time to reach the level M by the queue size, if starting from the level n, is equal to:*

(20) $$EH_{n,M}=\lim_{s\to 0+}{\left[R_{M}^{-1}\left(s\right)w_{M}\left(s\right)\right]}_{n},$$

*where ${\left[\cdot \right]}_{n}$ denotes the n-the element of a vector.*

In order to use Theorem 1 or Corollary 1 in practice, we have to be able to compute the values of $Q_{n,k}\left(u\right)$. Fortunately, $Q_{n,k}\left(u\right)$ has a known Laplace transform, namely:

(21) $$q_{n,k}\left(s\right)=\frac{\prod_{i=0}^{k-1}\lambda \left(1-d\left(n+i\right)\right)}{\prod_{i=0}^{k}\left(s+\lambda -\lambda d\left(n+i\right)\right)},\;\;\;\;\;\;\;n\geq 0,\;\;k\geq 0,$$

where

(22) $$q_{n,k}\left(s\right)=\int_{0}^{\infty }e^{-su}Q_{n,k}\left(u\right)du,\;\;\;s>0,$$

(see [21] for the proof).

Therefore, the values of $Q_{n,k}\left(u\right)$ can be computed using one of the available methods of inversion of the Laplace transform. In the following numerical examples, the Zakian method, [36], is used for this purpose. Namely, the original function G(t), given by its Laplace transform g(s), is equal approximately to:

(23) Gt≈2t∑k=04Reωkgαkt,

with coefficients $\alpha_{k}$ and $\omega_{k}$ given in Table 1:

The same inversion method can be used to invert (18) in order to obtain the probability that the queue size does not reach a given level.

## 4. Examples

In the examples, we focus on the impact of the shape and parameterization of the dropping function on the studied performance characteristics. Therefore, we assume simply that the arrival rate is 1, the service time is constant and equal to 1, and, as a consequence, ρ=1. We assume also that X(0+)=0. The assumption that initially the queue is empty is the most natural one, but all the calculations could be repeated for an arbitrary initial queue size, which is enabled by the formulas proven in the previous section.

**Figure 1:**
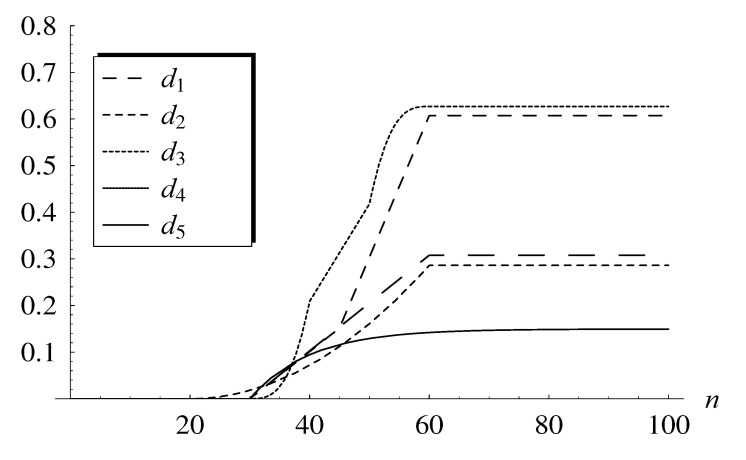
Dropping functions $d_{1}\left(n\right)-d_{5}\left(n\right)$.

The following five dropping functions are used in the examples (see also Figure 1):

- RED-type dropping function, [12]:
  (24) $$d_{1}\left(n\right)=\left\{\begin{array}{ccc} 0, & \mathrm{if} & n<30,\\ 0.01026n-0.30775, & \mathrm{if} & 30\leq n<60,\\ 0.30775, & \mathrm{if} & n\geq 60, \end{array}\right.$$
- GRED-type dropping function, [13]:
  (25) $$d_{2}\left(n\right)=\left\{\begin{array}{ccc} 0, & \mathrm{if} & n<30,\\ 0.01012n-0.30368, & \mathrm{if} & 30\leq n<45,\\ 0.03037n-1.21470, & \mathrm{if} & 45\leq n<60,\\ 0.60735, & \mathrm{if} & n\geq 60, \end{array}\right.$$
- NLRED-type dropping function, [15]:
  (26) $$d_{3}\left(n\right)=\left\{\begin{array}{ccc} 0, & \mathrm{if} & n<20,\\ 0.000178781{\left(n-20\right)}^{2}, & \mathrm{if} & 20\leq n<60,\\ 0.28605, & \mathrm{if} & n\geq 60, \end{array}\right.$$
- TRED-type dropping function, [17]:
  (27) $$d_{4}\left(n\right)=\left\{\begin{array}{ccc} 0, & \mathrm{if} & n<30,\\ 0.000208916{\left(\frac{n-30}{30}\right)}^{3}, & \mathrm{if} & 30\leq n<40,\\ 0.020892n-0.62675, & \mathrm{if} & 40\leq n<50,\\ 0.000208916{\left(\frac{n-60}{30}\right)}^{3}+0.62675, & \mathrm{if} & 50\leq n<60,\\ 0.62675, & \mathrm{if} & n\geq 60, \end{array}\right.$$
- REM-type dropping function, [14]:
  (28) $$d_{5}\left(n\right)=\left\{\begin{array}{ccc} 0, & \mathrm{if} & n<30,\\ 0.14950-0.14950e^{-(n-30)/10}, & \mathrm{if} & n\geq 30. \end{array}\right.$$

They represent the most popular dropping function classes proposed in the active queue management literature. Importantly, all these dropping functions were parameterized in [28] in such a way that they provide the average stationary response time of exactly 20.0 for ρ=1. The average response time is arguably the single most important stationary performance measure of a queueing system, thus in some sense, these five dropping functions provide the same stationary behavior of the queue.

As we will see, it does not mean that the transient behavior is similar as well. Namely, in Table 2, the probability of not reaching a queue size of level 50 in 1000 seconds is shown for functions $d{}_{1}$–$d{}_{5}$. It is accompanied by the average time to reach level 50. As we can see, the probability varies significantly, from 0.933 to 0.997, depending on the dropping function. The mean time to reach 50 varies even more—from about 8000 to over 200,000.

**Figure 2:**
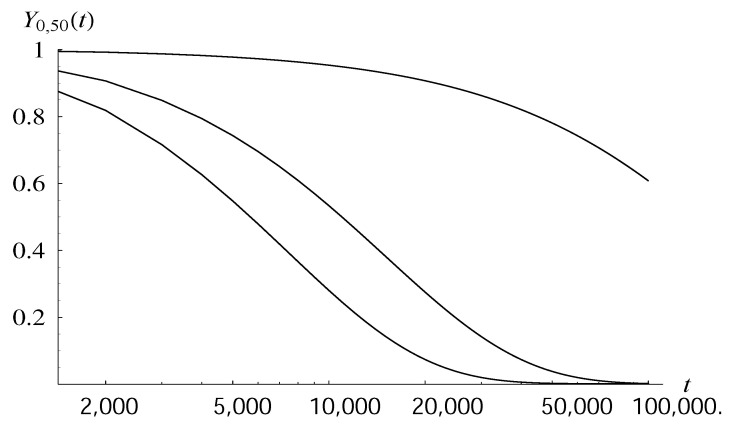
Probability of not reaching level 50 in interval (0,t) as a function of *t*, for dropping functions $d_{3}$, $d_{2}$, $d_{4}$, counting from the bottom.

The detailed dependence of the probability of not reaching 50 by the time *t* is depicted as a function of *t* in Figure 2, for three selected dropping functions. We see a very different transient performance in each case. For instance, probability 0.6 of not crossing 50 corresponds to the time interval of length about 8000 in the case of $d_{2}$, of length about 4300 in the case of $d_{2}$, and of length over 100,000 in the case of $d_{2}$.

**Figure 3:**
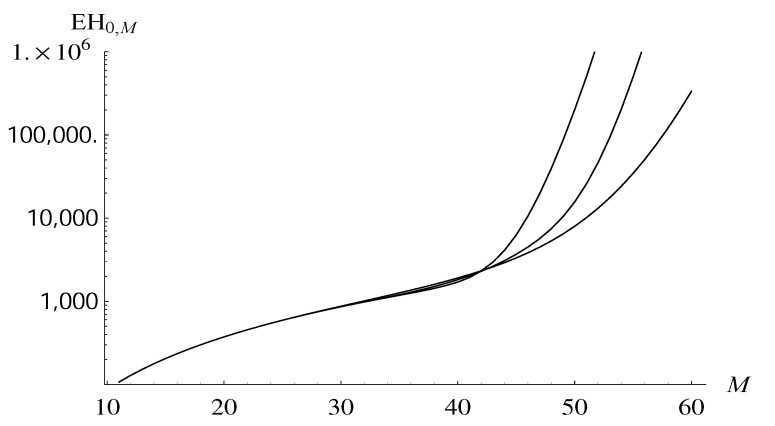
The average time to reach level *M* as a function of *M*, for dropping functions $d_{3}$, $d_{2}$, $d_{4}$, counting from the bottom.

In Figure 3, the average time to reach level *M* is depicted for the same three dropping functions, as a function of *M*. As we can see, for low *M* values, all three graphs are the same, which follows from the fact that all functions $d_{2}$-$d_{4}$ vanish for small *n*. From *M* about 35, however, the graphs start to diverge, and the divergence becomes very fast for *M* over 42 (note that the scale on the vertical axis is logarithmic).

All of these considerations indicate clearly, that the transient behavior of the system, may differ by far for different dropping functions, even if the stationary average response time is the same for all of them.

**Figure 4:**
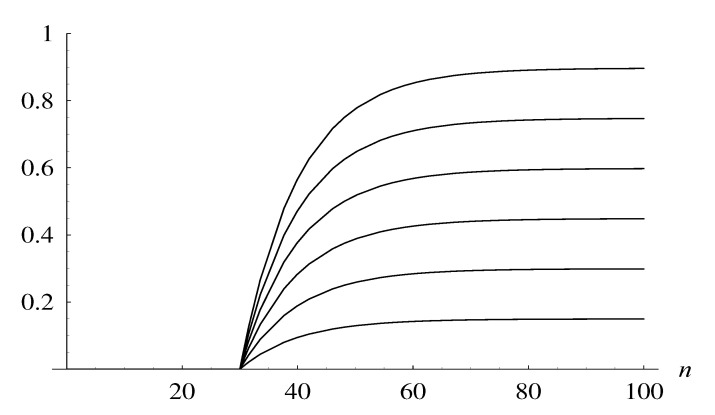
Dropping function d(p,n) for p=1,⋯,6, counting from the bottom.

In the final example, we show that we can design the dropping function in such a way that it meets some performance goals. For this purpose we can use, for instance, the following class of dropping functions:

(29) $$d\left(p,n\right)=pd_{5}\left(n\right),$$

where *p* is a positive parameter. Sample functions from this class are shown in Figure 4.

Assume now that we have the following performance goals: the probability that the queue size does not reach 50 in one hour (3600s) has to be at least 0.99, while the overall packet loss ratio has to be as small as possible. (The loss ratio is defined, naturally, as the long-run fraction of dropped packets).

Using the bisection method and Theorem 1, we can easily obtain the value of *p* for which it holds $Y_{0,50}\left(3600\right)=0.99$. It is p=2.237. Moreover, for every *n* we have $d\left(p_{1},n\right)\leq d\left(p_{2},n\right)$ if $p_{1}<p_{2}$. Thus it is obvious that the loss ratio must grow with *p*, when the class d(p,n) of dropping functions is used.

Therefore, we conclude that the optimal dropping function in the class (29), with respect to the assumed performance goals, is d(2.237,n).

## 5. Conclusions

We derived a formula for the probability that in a time interval of length *t*, the queue size is kept under a given level, in a queuing system with the dropping function. In addition, a formula for the average time to reach a given level was obtained. As the main reason to apply the dropping function mechanism is to maintain a low queue size, these are natural characteristics to study.

In numerical examples it was demonstrated that for a precise characterization of the system, stationary performance characteristics should be accompanied by the above-mentioned transient probabilities when the dropping function is applied. Namely, it was shown that different dropping functions may induce a very different transient behavior of the system, even if they provide the same stationary behavior (e.g., the average response time).

The study was motivated by active queue management in Internet routers. The results are presented, however, in universal terms of queueing theory, and are applicable in other areas as well. 

## Figures and Tables

**Table 1:** Coefficients for the Zakian inversion algorithm.

Coefficient	Value
$\alpha_{0}$	12.83767675 + *i*1.666063445
$\alpha_{1}$	12.22613209 + *i*5.012718792
$\alpha_{2}$	10.93430308 + *i*8.409673116
$\alpha_{3}$	8.776434715 + *i*11.92185389
$\alpha_{4}$	5.225453361 + *i*15.72952905
$\omega_{0}$	-36902.08210 + *i*196990.4257
$\omega_{1}$	61277.02524 − *i*95408.62551
$\omega_{2}$	28916.56288 + *i*18169.18531
$\omega_{3}$	4655.361138 − *i*1.901528642
$\omega_{4}$	−118.7414011 −*i*141.3036911

**Table 2:** Probability of not reaching 50 in (0,1000) and the average time to reach 50 for dropping functions $d_{1}-d_{5}$.

Dropp. Fun.	$Y_{0,50}\left(1000\right)$	$EH_{0,50}$
$d_{1}\left(n\right)$	0.96242	13,733
$d_{2}\left(n\right)$	0.96728	15,621
$d_{3}\left(n\right)$	0.93350	7973
$d_{4}\left(n\right)$	0.99746	200,534
$d_{5}\left(n\right)$	0.94178	10,002
